# The Association between Ranitidine Use and Gastrointestinal Cancers

**DOI:** 10.3390/cancers13010024

**Published:** 2020-12-23

**Authors:** Gerald McGwin

**Affiliations:** Department of Epidemiology, School of Public Health, University of Alabama at Birmingham, Birmingham, AL 35223, USA; gmcgwin@uabmc.edu

**Keywords:** ranitidine, gastrointestinal cancers, N-nitrosodimethylamine (NDMA)

## Abstract

**Simple Summary:**

N-nitrosodimethylamine (NDMA) is a carcinogen in experimental animals and has been classified a probable human carcinogen. NDMA has been identified in samples of ranitidine. Observational studies have demonstrated a relationship between dietary and occupational exposure to NDMA and specific cancers, principally to the gastrointestinal system. Studies focused on ranitidine exposure are lacking. This study evaluated the association between ranitidine exposure and gastrointestinal cancer using a comparison group that minimizes confounding by indication.

**Abstract:**

N-nitrosodimethylamine (NDMA) is a carcinogen in experimental animals. It has been classified a probable human carcinogen and has been found in ranitidine. This study sought to evaluate the association between ranitidine use and cancer of the gastrointestinal system. Events reported to the FDA Adverse Events Reporting System that were associated with the use of proton pump inhibitors (PPIs) and H_2_ antagonists were selected. Proportionate reporting ratios (PRRs) and associated 95% confidence intervals (CIs) were calculated to compare the proportion of all reported adverse events that were for gastrointestinal system cancers among adverse event reports for ranitidine to adverse event reports for other H_2_ antagonists. The proportion of adverse events for any gastrointestinal system cancer relative to all other events was elevated for ranitidine compared to PPIs and other H_2_ antagonists (PRR 3.66, 95% CI 3.19–4.20). Elevated and significant PRRs were observed for pharyngeal (PRR 9.24), esophageal (PRR 3.56), stomach (PRR 1.48), colorectal (PRR 16.31), liver (PRR 2.64), and pancreatic (PRR 2.18) cancers. The PRRs for anal (PRR 4.62) and gallbladder (PRR 4.62) cancer were also elevated though not statistically significant. In conjunction with a large body of epidemiologic and human and animal basic science research, the study results support the hypothesis that NDMA-contaminated ranitidine increases the risk of cancer and supports the withdrawal of these medications from the market.

## 1. Introduction

Ranitidine (Zantac^®^) is an H2 antagonist used to treat heartburn, stomach ulcers, gastroesophageal reflux disease (GERD), and other conditions associated with the overproduction of stomach acid. It was approved for use in the United States in 1983 and by 1988 it had become the world’s best-selling drug [1]. Ranitidine was approved for over-the-counter use in 2004 and, until recently, was sold as Zantac as well as private label and generic products. In 2019, N-nitrosodimethylamine (NDMA) was identified in samples of ranitidine which led the FDA to alert the public of the potential risks associated with NDMA exposure, which include cancer [2]. The FDA has demonstrated that NDMA levels in ranitidine increase under normal storage conditions and increase significantly under higher temperatures that may occur during distribution and handling [3]. They also found that the older the ranitidine product is or the longer the length of time since manufacturing, the higher the levels of NDMA. Given these findings, in April 2020, the FDA announced that ranitidine was to be withdrawn from the market and warned consumers to cease use of the product. 

Research has demonstrated that NDMA is a potent carcinogen in experimental animals and has been classified a probable human carcinogen [4]. While not currently produced in the United States for commercial purposes, NDMA it is a byproduct of certain industrial processes and can be released into the air, soil, and water as a result [5]. NDMA can also be formed naturally, typically via the consumption of certain food items. Human exposure to NDMA usually occurs via the diet, through the consumption of contaminated water and/or foods that contain nitrosamines (e.g., cured meat) or alkylamines (e.g., tea). Exposure can also occur via the use of NDMA-containing cosmetic products and in occupational settings. 

A large number of epidemiologic studies have evaluated the association between ranitidine use, NDMA exposure, and cancer. The majority of these studies have focused on cancers of the gastrointestinal system; of those studies, gastric cancer has received the most attention. In a meta-analysis, Song et al. reported that high levels of dietary NDMA exposure were associated with a statistically significant 34% increase in the risk of gastric cancer [6]. Occupational exposure to NDMA has been associated with stomach cancer in one study [7] but not another [8]. There has been a limited number of studies regarding the association between ranitidine use and gastrointestinal cancer [9,10,11,12]. Habel et al. investigated the association between prescription ranitidine use and specific cancers and reported a statistically significant 2.4-fold increased risk for gastric/esophageal cancer [9]. More recently, Iwagami et al. evaluated the risk of cancer among new users of ranitidine and nizatidine compared to H_2_ antagonists and found no association [12]. However, the duration of follow-up was perhaps too short to observe an association and over-the-counter medication use was not considered; therefore, misclassification of ranitidine use may have underestimated the true association. A number of studies have also investigated the relationship between dietary and occupational NDMA exposure as well as ranitidine use and other gastrointestinal cancers including liver [11], esophageal [13], pancreas [14], and colorectal cancer [15]. While the results of this body of research are heterogeneous, including positive, null, and negative associations, the majority of studies report positive associations, many of which are statistically significant. However, few of these studies have investigated ranitidine use specifically; therefore, this represents a significant gap in the literature given the current environment. The objective of the current study was to evaluate the association between ranitidine use and other non-NDMA contaminated proton pump inhibitors (PPIs) and H2 antagonists and gastrointestinal cancer. 

## 2. Results

For the period from 2013 to the first quarter of 2020, there were 637,563 mentions of PPIs and H2 antagonists in the FDA Adverse Event Reporting System (FAERS) representing 143,359 unique adverse event reports. Following the exclusion of duplicate adverse event reports and those mentioning ranitidine and at least one H_2_ antagonist or PPI (i.e., non-ranitidine monotherapy), a total of 141,963 reports were selected for the analysis. There were 13,856 (9.8%) adverse event reports for ranitidine monotherapy. Table 1 presents the medication, demographic, and reporting characteristics for the adverse event reports associated with ranitidine, as well as other PPIs and H2 antagonists. The majority of non-ranitidine adverse event reports were for PPIs, specifically omeprazole, esomeprazole, lansoprazole, and pantoprazole. The age and gender distribution of both groups was similar and consistent with the epidemiology of the conditions for which these drugs are used. For adverse event reports wherein a known indication was provided, for ranitidine the most common indication was dyspepsia (37.5%) followed by gastroesophageal reflux disease (21.2%) and a variety of specific gastrointestinal conditions (e.g., gastritis, gastric ulcer); for the non-ranitidine reports, the most common indication was gastroesophageal reflux disease (70.7%) followed by dyspepsia (7.7%). The number of adverse event reports has increased over time, with substantial increases for both groups beginning in 2018, recognizing that data for only one quarter of 2020 was included. 

Table 2 presents the number and proportion of ranitidine and other PPIs and H2 antagonist adverse event reports associated with digestive system cancers as well as the associated proportionate reporting ratios (PRRs), 95% CIs, and p-values. Overall, the proportion of adverse events for any gastrointestinal system cancer relative to all other events was elevated for ranitidine compared to PPIs and H_2_ antagonists (PRR 3.66, 95% CI 3.19–4.20). Elevated and significant PRRs were observed for pharyngeal, esophageal, stomach, colorectal, liver, and pancreatic cancers; the PRRs for anal and gallbladder cancer were also elevated. 

## 3. Discussion

The results of the current study supported the interpretation that NDMA is carcinogenic in humans, specifically to the gastrointestinal system. This study was unique in that it compared cancer occurrence among ranitidine users to that of a population that uses medications to treat similar conditions, though not known to be contaminated with NDMA. Prior studies have compared ranitidine users to non-users [9,10,11]; the latter likely having a low prevalence of the underlying conditions for which H_2_ antagonists and PPIs are taken, but are also associated with an increased cancer risk. By comparing ranitidine users to users of PPIs and other H_2_ antagonists, the current study was better able to reduce the impact of confounding by indication and thereby isolate the role of NDMA. Using a similar design, Iwagami et al. reported no significant or elevated associations for a wide range of cancers; however, the results of this study were subject to several important limitations including the short duration of follow-up and misclassification, which likely resulted in underestimates of the true associations [12]. 

Human NDMA exposure can occur exogenously via the consumption of contaminated foods or breathing contaminated air or endogenously via the transformation of nitrosamine precursors. The metabolism of NDMA and mechanism by which it induces tumors is well understood and is believed to be the same for both animals and humans [16]. Thus, the relationship between NDMA exposure and gastrointestinal cancer is biologically plausible; in fact, the evidence for this relationship is substantial. A detailed discussion of the mechanism by which NDMA exposure induces cancer was beyond the scope of the current manuscript, though this information is available elsewhere [17,18]. Briefly, the formation (in the case of endogenous NDMA), absorption, distribution, metabolism, and excretion of NDMA primary involves the organs of the gastrointestinal system, thereby placing them at risk for cancer induction via exposure to pathways that cause DNA damage [19]. 

The FDA has determined that NDMA is one of seven nitrosamine impurities that may be present in drugs, including ranitidine [3]. The root causes of this contamination occur during the manufacture and storage process. Nitrosamines have been classified as probable or possible human carcinogens by the International Agency for Research on Cancer [19]. NDMA has specifically been classified as a probable human carcinogen assumed to be positively associated with stomach and colorectal cancer based on the results of epidemiologic research. The World Health Organization has likewise acknowledged the carcinogenicity of NDMA even at low levels of exposure based upon considerable evidence from in vivo, in vitro, and epidemiologic research [4]. Tumor formation, specifically esophageal and hepatic, in rats exposed to NDMA at doses of approximately 10 µg/kg/day has been observed [20]. NDMA levels of 2.5 million nanograms (i.e., 2500 µg) have been observed in samples of various ranitidine products [21]. At such levels, the daily exposure for a 70-kg human would be approximately 35 µg/kg/day, three times higher than the daily dose observed to produce tumors in rats. The FDA tested a range of ranitidine-containing products and found NDMA in all of the samples that tested, though at lower levels than reported previously [2]. The highest reported level across all products tested was 0.86 µg, with many products having levels of 0.01 µg. However, others have reported much higher NDMA levels (500 ppm) in ranitidine-containing products under certain storage conditions [22]. For reference, the FDA considers consuming up to 0.096 µg (0.32 ppm) of NDMA per day reasonably safe based on lifetime exposure. The reasons behind the wide variation in NDMA levels observed in ranitidine-containing products are unclear, but may be attributable to differences in testing protocols. 

The results of the current study are consistent with the large number of epidemiologic studies evaluating the association between NDMA exposure and gastric cancer. In a meta-analysis of dietary exposure to nitrates, nitrites, and nitrosamines, the relative risk of stomach cancer for the highest levels of NDMA exposure compared to the lowest was 1.34 [6]. Two studies have evaluated the cancer mortality experience of rubber workers occupationally exposed to NDMA, reporting standardized mortality ratios of 1.2 and 1.72, the latter being statistically significant [7,8]. Habel et al. estimated the relative risk (RR) for specific cancers associated with ranitidine use among members of a large health maintenance organization [9]. Ranitidine use was associated with a statistically significant 2.4-fold increased risk for the combined outcome of stomach/esophageal cancer. However, Iwagami et al. reported similar risks for gastric cancer among new users of prescription ranitidine and/or nizatidine and other H_2_ antagonists [12]. This study did not take into account over-the-counter medication use, which likely biased the association towards the null. The stronger association for esophageal cancer (PRR = 3.56) observed in the current study was also consistent with existing work. Studies of dietary NDMA exposure are equivocal, with three reporting significant positive associations indicating at least a two-fold increased risk [13,23,24] and three reporting non-significant weaker associations [13,15,25]. Two occupational studies both reported significant associations with standardized mortality ratios (SMRs) of 9.1 and 3.0 [7,8]. In a case-control study of males with Barrett’s esophagus, Tan et al. reported a protective effect of ranitidine use but for those with lower defined daily doses (DDD), a three-fold increase risk was observed; for higher DDD, there was a strong protective effect [10]. 

The strongest association observed in the current study was for colorectal cancer. Research regarding NDMA exposure and colorectal cancer has been equivocal. Of the three studies evaluating dietary exposure, two reported significant positive associations, while the third only reported a significant positive association for rectal, but not colon, cancer [15,25,26], whereas two occupational studies and studies evaluating ranitidine use did not report significant associations [7,8,9,12]. 

The liver plays a prominent role in the metabolism of nitrosamines, and hepatic tumors have been linked to NDMA exposure in animal studies; thus, the elevated PRR for liver cancer observed in the current study was not surprising. However, with one exception, prior studies have failed to document a significant association between NDMA exposure or ranitidine use and liver cancer. Hidjat et al. observed a statistically significant dose–response relationship between occupational NDMA exposure and liver cancer mortality [7]. In contrast, Straif et al. observed no association in a similar occupational cohort [8]. Tran et al. evaluated the association between PPIs and H_2_ antagonists and the risk of liver cancer in two population-based analyses [11]. Both sets of analyses revealed increased risks for ranitidine compared to non-users (41% and 82%), though neither association was statistically significant. 

The positive association between ranitidine and pancreatic cancer observed in the current study was consistent with the results reported by Habel et al., who reported a statistically significant 2.6-fold increased risk associated with ranitidine use [9]; however, Iwagami et al. did not [12]. Hidajat et al. also found a statistic association between occupational NDMA exposure and deaths due to pancreatic cancer, though Straif et al. did not [7]. In a case-control study of pancreatic cancer and dietary exposure to N-nitroso compounds, Zheng et al. found no association between overall NDMA exposure; however, for NDMA exposure due to plant-based foods, a dose–response relationship was observed. No such association was observed for NDMA from animal-based foods [14]. 

For the remaining cancers investigated in the current study, contextualizing the results was difficult due to the paucity of prior literature. Straif et al. reported elevated SMRs for oral and pharyngeal cancer, which was consistent with the elevated PRR for pharyngeal cancer in the current study, but inconsistent with the lack of an association with oral cancer [8]. These authors combined liver and gallbladder cancer into a single entity and reported no association, whereas elevated PRRs were observed for both organs in the current study. Similarly, the combined outcome of anal and rectal cancer demonstrated no association, which is in contrast to the current findings. However, in their investigation of NDMA and colorectal cancer, Zhu et al. reported site-specific results for the proximal and distal colon and rectum [26]. The results indicated a statistically significant positive trend between dietary NDMA exposure and rectal cancer. Loh et al. similarly reported a positive association [25]. 

The results of the current study should be interpreted in light of several limitations, primarily stemming from the well-known limitations of the FAERS data. The FAERS is based on spontaneous reports of adverse events; it does not purport to represent all occurrences of adverse drug events. However, this is not a limitation unique to the FAERS; in fact, it would be rare for any epidemiologic study to enumerate all occurrences of an outcome under investigation. Rather than complete enumeration, epidemiologic studies focus on identifying outcomes in an unbiased manner. There was little reason to suspect any such bias in the current study as the majority of reports were submitted prior to media reports of NDMA-contaminated ranitidine drugs. Further, research suggests that such notoriety bias does not lead to over-reporting in the FAERS and has minimal impact on measures of association [27]. All information was self-reported and not subject to validation, and a causal relationship between the reported medication(s) and adverse event(s) in any given report could not be inferred. Though information on the validity of self-reported use of H_2_ antagonists and PPIs was lacking, Tran et al. conducted a pair of studies on the relationship between PPIs and H_2_ antagonists and the risk of liver cancer [11]. In one study, medication use was obtained via self-report, whereas in the other, prescription records were used. The associations reported in each study were similar, lending support for the validity of the self-reported medication use. Unlike a cohort study, the lack of a true denominator (i.e., the number of persons prescribed or who use a product) precluded the calculation of incidence rates. However, the calculation of reporting ratios (PRR, POR) from pharmacovigilance data as valid estimates of relative risks has been described and their use is widely accepted [28,29]. Further, the results of disproportionality analyses of the FAERS data has been shown to yield findings that are consistent with the results of population-based studies [30,31,32,33,34]. 

## 4. Materials and Methods

### 4.1. Data Source

This study was reviewed by the University of Alabama at Birmingham Institutional Review Board for Human Use and deemed not human subject research given the public availability and de-identified nature of the data source. 

The data for this study were obtained from the United States FDA Adverse Event Report System (FAERS), which is a database containing adverse event reports, medication error reports, and product quality complaints that result in adverse events. FAERS is used by the FDA and other entities for the post-marketing surveillance of medications and biologics. It is also used for research purposes. Adverse event reports are voluntarily submitted to the FAERS by a wide range of entities including healthcare professionals, consumers, lawyers, and drug manufacturers. Drug manufacturers who receive adverse event reports from health professionals and consumers must in turn submit them to the FDA. Individual adverse event reports can contain information on one or more drugs and/or adverse events. In the case of multiple adverse events, there is no hierarchy with respect to severity or importance. However, each drug is classified as a “primary suspect drug”, “secondary suspect drug”, “concomitant”, or “interacting”. Adverse event reports may be incomplete, containing missing information for individual data fields. 

The FAERS data is publicly available and is updated quarterly. The data for this study covered data for the period from 2013 through the first quarter of 2020. These updates included both newly submitted adverse event reports as well as updates to previously submitted reports. Updated reports may have included updates to previously submitted information or new information that was previously omitted. The identification of updated reports was facilitated by the use of a unique case identification number. When analyzing FAERS data, the FDA recommends conducting an initial de-duplicating process by selecting the most recently updated record for each unique case identification number; this process was utilized in the current study. The publicly available FAERS data are composed of seven individual data files corresponding to (1) patient demographic and administrative information; (2) drug/biologic information; (3) adverse event information; (4) patient outcome information; (5) adverse event reporting source; (6) drug/biologic therapy information (e.g., drug start date); and (7) indications for use of the reported drugs. With respect to drug information, the name of the product appears verbatim; therefore, any given drug may be identified by its trade or generic name, with and without misspellings and ancillary text strings (e.g., “Omeprazole Mylan (Non-AZ Product)”). Adverse events and indications are coded based on the Medical Dictionary for Regulatory Activities (MedDRA) preferred term level terminology. 

### 4.2. Drug Identification

For the purposes of the current study all adverse event reports for H_2_ antagonists and PPIs were selected using the following terms: “ZANTAC”, “RANITIDINE”, “PEPCID”, “FAMOTIDINE”, “TAGAMET”, “CIMETIDINE”, “AXID”, “NIZATIDINE”, “PRILOSEC”, “ZEGERID”, “OMEPRAZOLE”, “PREVACID”, “LANSOPRAZOLE”, “PROTONIX”, “PANTOPRAZOLE”, “NEXIUM”, “ESOMEPRAZOLE”, “ACIPHEX”, “RABEPRAZOLE”, “DEXILANT”, “DEXLANSOPRAZOLE”. (All drug names in the FAERS data files were first converted to upper case to facilitate the search algorithm.) Only primary and secondary suspected drugs were selected; drugs identified as concomitant or interacting were excluded. Adverse event reports that included the use of ranitidine and any other H_2_ antagonist or any PPI were also excluded. Non-ranitidine adverse event reports that included the use of multiple PPIs and/or H_2_ antagonists were retained. 

### 4.3. Adverse Event Identification

For the aforementioned PPI and H_2_ antagonist adverse event reports, all cancer-related adverse events were identified using the non-hematological malignant tumors Standardized MedDRA Query (SMQ). These preferred terms were then used to identify gastrointestinal system cancers, specifically: Oral cavity, pharynx, esophagus, stomach, small intestine, large intestine (including rectum), and anus. Cancers associated with the liver, gallbladder, and pancreas were also identified. 

### 4.4. Statistical Analysis

Adverse events reports associated with ranitidine were compared to those for other H_2_ antagonists and PPIs with respect to demographic, reporting, medication, and event characteristics. Proportionate reporting ratios (PRRs) and associated 95% confidence intervals (CIs) were calculated to compare the proportion of all reported adverse events that were for gastrointestinal system cancers among adverse event reports for ranitidine to adverse event reports for other H_2_ antagonists. Chi-square and, where appropriate, Fisher’s exact tests were also computed. *p*-values of ≤ 0.05 (two-sided) were considered statistically significant. 

## 5. Conclusions

The results of the current study provided direct support for the assertion that NDMA contaminated ranitidine is associated with the occurrence of gastrointestinal cancer. This assertion was bolstered by an abundance of evidence demonstrating that NDMA exposure is associated with an increased risk of cancer in humans as well as animals. In order to more fully understand the nature of this relationship, future observational studies should address issues related to the magnitude and duration of exposure, informed by research regarding NDMA levels in ranitidine products. 

## Figures and Tables

**Table 1:** Characteristics of adverse event reports for ranitidine and proton pump inhibitors (PPIs) and other H_2_ antagonists.

Adverse Event Report Characteristic	Ranitidine (N = 13,856)		PPIs and Other H_2_ Antagonists (N = 128,107)	
	Number	Percent	Number	Percent
**Type of H_2_ Antagonist/PPI**				
Ranitidine	13,856	100.0		
Famotidine			3630	2.6
Cimetidine			550	0.4
Nizatidine			342	0.2
Omeprazole			68,103	48.0
Esomeprazole			61,153	43.1
Lansoprazole			44,210	31.1
Pantoprazole			43,436	30.6
Dexlansoprazole			20,039	14.1
Rabeprazole			7783	5.5
**Age**				
<18	668	9.2	1883	2.6
18–64	3559	49.2	38,229	52.3
>64	3006	41.6	33,007	45.1
**Gender**				
Female	7933	66.7	60,657	61.5
Male	3957	33.3	38,044	38.5
**Initial Report Year**				
<2012	76	0.6	4268	3.3
2013	1013	7.3	7874	6.2
2014	1140	8.2	8423	6.6
2015	1367	9.9	10,525	8.2
2016	1665	12.0	11,699	9.1
2017	1183	8.5	10,813	8.4
2018	2804	20.2	32,850	25.6
2019	3706	26.8	36,692	28.6
2020	902	6.5	4963	3.9
**Reporting Entity**				
Consumer	9330	68.4	44,620	43.5
Physician	1697	12.4	16,241	15.9
Other health professional	1915	14.0	1833	17.9
Lawyer	21	0.2	16,056	15.7

**Table 2:** Number and proportion of digestive system cancer-related adverse event reports for ranitidine and PPIs and H_2_ antagonists and associated proportional reporting ratios and 95% confidence intervals.

Type of Cancer	Ranitidine (N = 13,856)	PPIs and Other H_2_ Antagonists (N = 128,107)	Proportional Reporting Ratio (95% Confidence Interval)	*p*-Value
	**N (%)**	**N (%)**		
**All**	282 (2.00)	712 (0.56)	3.66 (3.19–4.20)	<0.0001
**Mouth**	3 (0.02)	34 (0.03)	0.82 (0.25–2.66)	1.00
**Pharynx**	3 (0.02)	3 (0.00)	9.24 (1.87–45.80)	0.01
**Esophagus**	47 (0.34)	122 (0.10)	3.56 (2.54–4.98)	<0.0001
**Stomach**	46 (0.33)	288 (0.22)	1.48 (1.08–2.01)	0.02
**Small intestine**	1 (0.01)	9 (0.01)	1.03 (0.13–8.11)	1.00
**Colon/rectum**	157 (1.13)	89 (0.07)	16.31 (12.58–21.14)	<0.0001
**Anus**	1 (0.01)	2 (0.00)	4.62 (0.42–50.98)	0.27
**Gallbladder**	2 (0.01)	4 (0.00)	4.62 (0.85–25.24)	0.11
**Liver**	30 (0.22)	105 (0.08)	2.64 (1.76–3.96)	<0.0001
**Pancreas**	20 (0.14)	85 (0.07)	2.18 (1.34–3.54)	0.004
